# Regional and hospital variations in the extent of decline in the proportion of percutaneous coronary interventions performed for nonacute indications – a nationwide population-based study

**DOI:** 10.1186/s12872-017-0592-4

**Published:** 2017-06-09

**Authors:** Fu-Wen Liang, Tsung-Hsueh Lu, Hsin-Min Wu, Jo-Chi Lee, Wei-Hsian Yin

**Affiliations:** 1 The NCKU Research Center for Health Data and Department of Public Health, National Cheng Kung University, No. 1, University Road, East District, Tainan, 70101 Taiwan; 2 Division of Cardiology, Cheng Hsin General Hospital, No. 45, Cheng Hsin Street, Bei-Tou, Taipei, 11220 Taiwan; 3 School of Medicine, National Yang Ming University, No.155, Sec.2, Linong Street, Taipei, 11221 Taiwan

**Keywords:** Indication, Patient selection, Percutaneous coronary intervention, Utilization

## Abstract

**Background:**

The volume and percentage of percutaneous coronary interventions (PCIs) performed for nonacute indications have declined in the United States since 2007. However, little is known if similar trends occurred in Taiwan.

**Methods:**

We used data from Taiwan National Health Insurance inpatient claims to examine the regional and hospital variations in the extent of decline in the percentage of nonacute indication PCIs from 2007 to 2012.

**Results:**

The volume of total PCIs persistently increased from 29,032 in 2007 to 35,811 in 2010 and 37,426 in 2012. However, the volume of nonacute indication PCIs first increased from 7916 in 2007 to 9143 in 2009 and then decreased to 8666 in 2012. The percentage of nonacute indication PCIs steadily decreased from 27% in 2007 to 26% in 2009 and then to 23% in 2012, a − 15% change. The extent of decline was largest in the North region (from 27% to 21﻿%, a − 22% change) and least in Kaopin region (from 20% to 18%, a − 13% change). Of the 71 hospitals studied, 14 did not show a decreasing trend. Five of the 14 hospitals even showed an increasing trend, with a percentage change >10% between 2007 and 2012. In 2012, 6 hospitals had a nonacute indication PCI percentage >35%.

**Conclusions:**

In Taiwan, four-fifths of the hospitals showed a decline in the percentage of nonacute indication PCIs from 2007 to 2012. It is plausible that Taiwanese cardiologists would have been influenced by the recommendations of crucial US trials and guidelines.

## Background

After the publication of the Clinical Outcomes Utilizing Revascularization and Aggressive Drug Evaluation (COURAGE) trial in 2007 and the Appropriate Use Criteria for Coronary Revascularization (AUC) in 2009 [1, 2], the volume and percentage of percutaneous coronary interventions (PCIs) performed for nonacute indications (or stable ischemic heart disease, SIHD) declined prominently in the United States [3–10]. However, only few studies have further examined the regional and hospital variations in the percentage of PCIs for nonacute indications [5, 9, 10].

Mohan et al. suggested that among hospital referral regions, the variations in the rate of PCIs performed for SIHD declined by 28% after the publication of the COURAGE study, but geographic variations remained higher for SIHD than for acute coronary syndrome [5]. Two studies have revealed that although the overall proportion of nonacute PCIs classified as inappropriate has declined, the hospital-level variations in inappropriate PCIs persist [9, 10].

In the era of globalization and evidence-based medicine, crucial trials and guidelines published in the United States might also have a substantial impact on cardiologists worldwide. However, little is known regarding whether similar trends exist in Taiwan, a country which is on the other side of Pacific Ocean and which has a different healthcare-financing system from that of the United States. In this study, we sought to examine regional and hospital variations in the extent of decline in the proportion of PCIs performed for nonacute indications in Taiwan from 2007 to 2012. Furthermore, professional organizations and insurance payers are eager to know the number and types of hospitals that did not show the expected decreasing trend after the publication of an important trial and guideline in which the assessment of the appropriateness of performed PCIs is considered necessary.

## Methods

### Data sources

Taiwan National Health Insurance (NHI) inpatient claims data were used for this study. The NHI program was introduced in 1995 and covers more than 99% of the population of Taiwan. Because all patients receiving PCIs in Taiwan were required to be hospitalized for observation, we assessed only the inpatient claims data and not the outpatient claims data. The inpatient claims data comprised data on diagnosis and procedure codes, various service fees, patient characteristics, attending physicians, and hospitals (level of accreditation and ownerships) [11, 12]. This study was approved by the Institutional Review Board of Cheng Hsin General Hospital (CHGH-IRB No. (424)103-1).

### PCIs performed

We used International Classification of Disease, Ninth Revision, Clinical Modification (ICD-9-CM) procedure codes 36.01, 36.02, 36.05, 36.06, and 36.09, which were recorded in 5 of the discharge procedure columns in the NHI inpatient claims data between January 2007 and December 2012, to identify patients receiving PCIs. Taiwan NHI does not use ICD-9-CM codes 00.66, 36.00, and 36.07 for PCIs, which are used in US hospitals.

### Nonacute indications

Crucial information (such as the symptoms and functional assessment of patients and the results of stress testing) required to assess the indications for performed PCIs is not available in the claims data. We could use only the ICD-9-CM codes of 5 discharge diagnoses recorded in the claims data to determine the indications for performed PCIs. The diagnoses classified as acute indications were modified based on the study of Chan et al. [13]: 1) “ST-segment elevation myocardial infarction (STEMI)” if the ICD-9-CM code 410.0, 410.1, 410.2, 410.3, 410.4, 410.5, 410.6, or 410.8 was recorded; 2) “non-ST-segment elevation myocardial infarction (NSTEMI)” if the ICD-9-CM code 410.7 or 410.9 was recorded; 3) “unstable angina” if the ICD-9-CM code 411, 411.0, 411.1, 412, 413.0, 413.1, 414.02, 414.03, 414.04, 411.81, or 411.89 was recorded; 4) “heart failure” if the ICD-9-CM code 428.xx was recorded; 5) “ventricular arrhythmia” if the ICD-9-CM code 427.1 or 427.4 was recorded; and 6) PCI or coronary artery bypass grafting (CABG) surgery received during previous hospitalizations. PCIs performed in the absence of the aforementioned diagnoses were classified as those performed for nonacute indications.

### Statistical analyses

First, we determined the number of total and nonacute indication PCIs performed from 2007 to 2012 by region. The six regions defined in this study are similar to hospital referral regions in the United States. In each region, there are several tertiary referral medical centers and people live in the region usually will receiving PCI in hospitals within the region. Second, we used the percentage change [(2007-2012)*100/2007%] between 2007 and 2012 to indicate the extent of decline in the proportion of PCIs for nonacute indications. A box-and-whisker plot was used to present the regional and hospital variations in the extent of decline. Third, we assessed not only the extent of decline but also the absolute number and percentage of nonacute indication PCIs performed in the latest available year (ie, 2012 for this study). This information is key to determining the priority for further assessment or intervention.

Fourth, we compared the characteristics of hospitals with a different extent of decline in the percentage of nonacute indication PCIs. The cut points of tertile of percentage change of 71 hospitals was −30% and −6%. Hospitals with a percentage change of lower than −30% were classified as the largest decline group, between −30% and −6% as the moderate decline group, and higher than −6% as the least decline group. The hospital characteristics were the accreditation level (tertiary referral medical center vs. secondary referral hospital), ownership (public vs. private) volume of PCIs performed in 2007, and the location (region). The chi-square test was used to examine if the distribution of characteristics differed by the extent of decline. If the number of hospitals in one cell was less than 5, Fisher’s exact test was used.

Fifth, to examine if the decline of nonacute indication was associated the higher use of coronary angiography. Pearson correlation was used to examine the association between hospital percentage change in number of coronary angiography between 2007 and 2012 and hospital percentage change in decline of nonacute indication PCIs.

## Results

A total of 205,834 PCIs were performed in Taiwan between January 2007 and December 2012. The volume of total PCIs performed persistently increased from 29,032 in 2007 to 35,811 in 2010 and 37,426 in 2012 (Table 1). However, the volume of nonacute indication PCIs first increased from 7916 in 2007 to 9143 in 2009 and then decreased to 8666 in 2012. The percentage of nonacute indication PCIs persistently decreased from 27% in 2007 to 26% in 2009 and then 23% in 2012.

**Table 1:** Number and percentage (%) of nonacute PCIs from 2007 to 2012 in Taiwan by region

	2007	2008	2009	2010	2011	2012	% change*
Taiwan							
Non-acute PCI	7916	8420	9143	8725	8519	8666	9.5
Total PCI	29,032	32,074	35,219	35,811	36,272	37,426	28.91
%	27.3	26.3	26.0	24.4	23.5	23.2	−15.1
Taipei							
Non-acute PCI	3497	3679	4154	3780	3910	3773	7.9
Total PCI	10,756	11,441	12,822	12,567	12,985	12,905	20.0
%	32.5	32.2	32.4	30.1	30.1	29.2	−10.1
North							
Non-acute PCI	845	783	866	965	898	847	0.2
Total PCI	3133	3260	3723	3935	3851	4033	28.7
%	27.0	24.0	23.3	24.5	23.3	21.0	−22.1
Central							
Non-acute PCI	1533	1677	1716	1699	1584	1710	11.5
Total PCI	5617	6575	6739	7108	7139	7472	33.0
%	27.3	25.5	25.5	23.9	22.2	22.9	−16.1
South							
Non-acute PCI	1144	1214	1394	1310	1131	1179	3.1
Total PCI	4793	5218	6072	6082	5731	6001	25.2
%	23.9	23.3	23.0	21.5	19.7	19.6	−17.7
Kaopin							
Non-acute PCI	780	929	900	811	849	1036	32.8
Total PCI	3900	4489	4816	4961	5516	5930	52.1
%	20.0	20.7	18.7	16.3	15.4	17.5	−12.6
East							
Non-acute PCI	117	138	113	160	147	121	3.4
Total PCI	833	1091	1047	1158	1050	1085	30.3
%	14.0	12.6	10.8	13.8	14.0	11.2	−20.6

* % change = (2012-2007)*100/2007

Regarding regional variation, in 2007, the percentage ranged from 19% in the Kaopin and East region to 33% in the Taipei region. In 2012, the percentage ranged from 18% in the Kaopin region to 29% in the Taipei region (Table 1). The extent of decline was largest in the North region (from 27% in 2007 to 21% in 2012, a − 22% change) and least in the Kaopin region (from 20% in 2007 to 18% in 2012, a − 13% change).

**Fig. 1:**
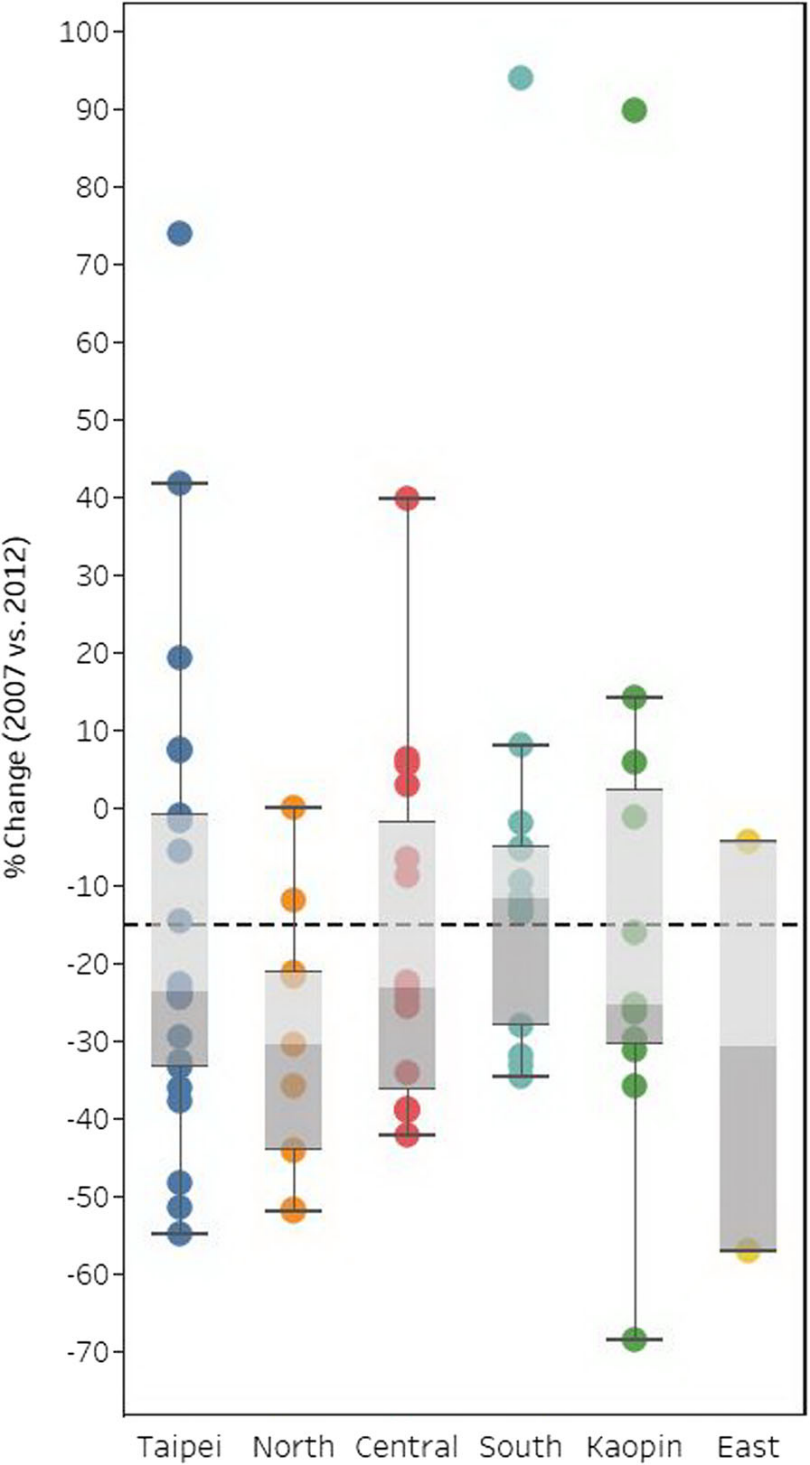
Box-and-whisker plot of hospital variation in extent of decline in nonacute PCIs from 2007 to 2012 in Taiwan by region (size of the circle indicates the volume of PCIs performed in each hospital in 2012)

Regarding hospital variation, we studied only 71 hospitals that performed at least 30 PCIs per year during the study period. There were two hospitals in 2007 and four hospitals in 2012 the annual PCIs performed was less than 30. Of the 71 hospitals, 57 showed a decreasing trend (i.e., the percentage change between 2007 and 2012 was negative; Fig. 1). Of the 14 hospitals that did not show a decreasing trend, 5 had a percentage difference of larger than 10% between 2007 and 2012. The interquartile range of percentage change between 2007 and 2012 was −0.9% to −33.4% in the Taipei region, −21.3% to −44.2% in the North region, −1.9% to −36.4% in the Central region, −5.0% to −28.0% in the South region, and 2.3% to −30.5% in the Kaopin region, and −4.4% to −57.1% in the South region. All hospitals in the North region showed decreasing trends. The hospital variation in the extent of decline was smallest in the South region.

**Fig. 2:**
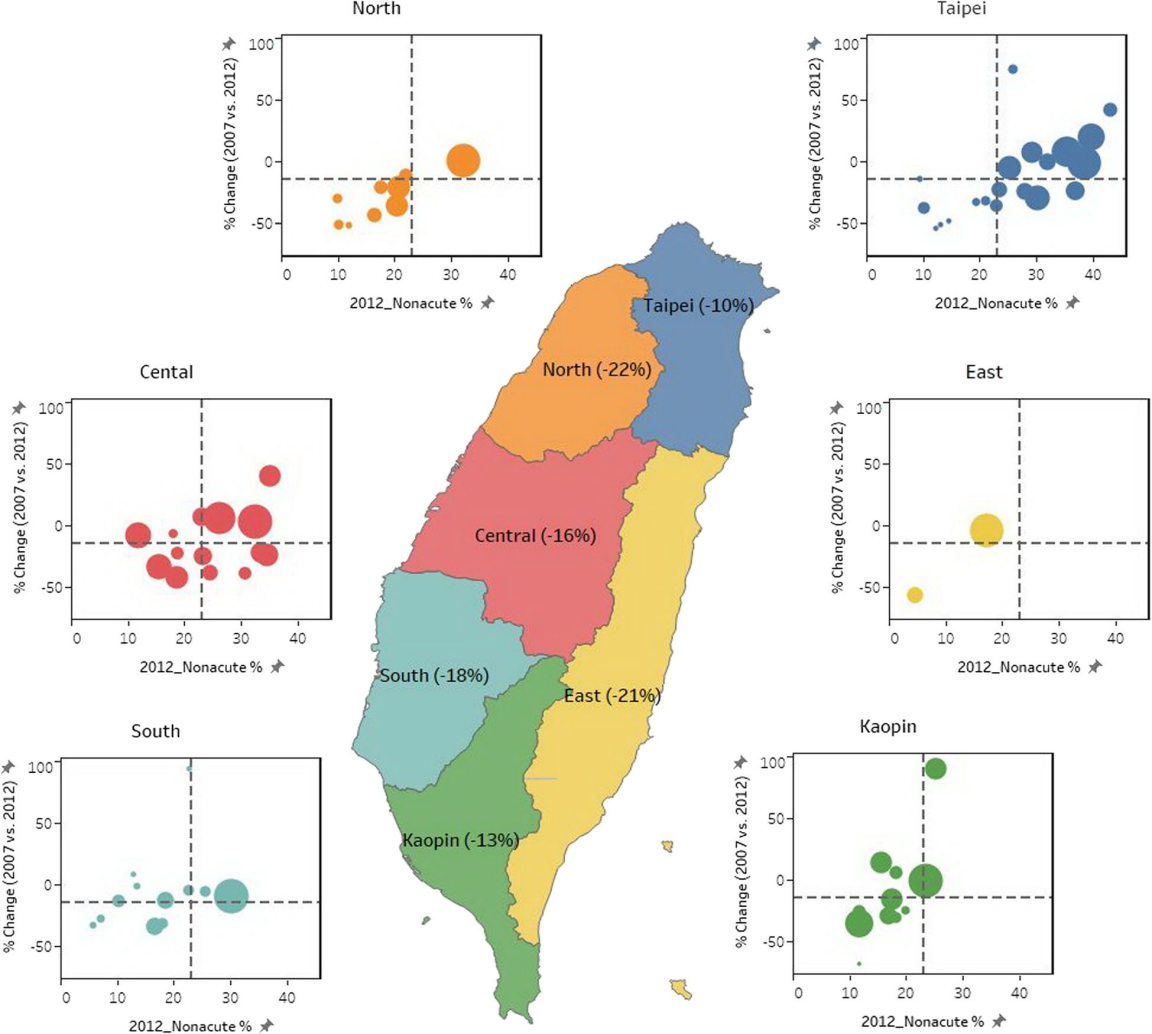
Scatter bubble plots of the hospital percentage change (2007 vs. 2012) and the percentage of nonacute PCIs in 2012 in Taiwan by region (size of the circle indicates the volume of nonacute PCIs in each hospital in 2012 and vertical and horizontal dot lines indicate the value of Taiwan)

In Fig. 2, we present both the percentage change between 2007 and 2012 and the percentage of nonacute indication PCIs in 2012 by hospital and region. The percentage of nonacute indication PCIs in 2012 was higher than 35% for 6 hospitals, and 5 of these hospitals were in the Taipei region and had a relatively large volume of performed PCIs. Furthermore, in 4 hospitals, the percentage of nonacute indication PCIs was between 30% and 35% in 2012, and these hospitals conducted more than 250 nonacute indication PCIs.

Despite no significant difference in the characteristics of hospitals with variations in the extent of decline, secondary referral hospitals showed the largest extent of decline compared with their counterpart hospitals (Table 2). No significant association between percentage change of performing coronary angiography between 2007 and 2012 and percentage change of nonacute indication PCIs between 2007 and 2012.

**Table 2:** Characteristics of hospitals with different extents of decline in nonacute PCIs from 2007 to 2012 in Taiwan

	Total		Largest decline		Moderate decline		Least decline		
	No	%	No	%	No	%	No	%	*p* value*
Level of hospital									0.368
Tertiary referral medical center	17	24	4	17	5	21	8	35	
Secondary referral hospital	54	76	20	83	19	79	15	65	
Ownership of hospital									0.762
Public	21	30	6	25	7	29	8	35	
Private	50	70	18	75	17	71	15	65	
Volume of total PCI in 2007									0.959
High (>384)	28	39	8	33	10	42	10	43	
Moderate (170-384)	22	31	8	33	7	29	7	30	
Low (<170)	21	30	8	33	7	29	6	26	
Region									0.900
Taipei	21	30	7	29	6	25	8	35	
North	9	13	5	21	3	13	1	4	
Central	15	21	5	21	6	25	4	17	
South	13	18	3	13	5	21	5	22	
Kaupin	10	14	3	13	4	17	4	17	
East	2	3	1	4	0	0	1	4	
Total	71	100	24	100	24	100	23	100	

*Chi-square test

## Discussion

The findings of this study indicate that despite the persistent increase in the volume of total PCIs performed from 2007 to 2012 in Taiwan, the percentage of PCIs performed for nonacute indications steadily decreased from 2007 to 2012, after the publication of the COURAGE trial in 2007 and the AUC guideline in 2009 in the United States. Approximately four-fifths of the studied hospitals showed a decreasing trend in the percentage of nonacute indication PCIs. However, a small number of hospitals still showed an increasing trend and a relatively high percentage of nonacute indication PCIs in 2012.

The persistent increase in the volume of total PCIs performed from 2007 to 2012 in Taiwan was due to the surge in the number of hospitals offering PCIs as a response to the government-initiated Door-to-Balloon Alliance in 2008, which was introduced to improve the survival rate of patients with STEMI, especially in remote areas [11]. Furthermore, the overall PCI utilization rate in Taiwan was 2056 per million adults in 2012, which was almost half of the utilization rate in the United States (3667 per million adults in 2007-2008) [14]. The momentum of increase in the PCI utilization rate in Taiwan may not yet have reached the peak. In addition, even studies in Korea (from 2006 to 2010) and Ireland (from 2004 to 2011) did not show any signs of decrease in PCI utilization [15, 16].

Although the volume of total PCIs persistently increased from 2007 to 2012, we noted a decrease in the volume and percentage of nonacute indication PCIs from 2009 and 2007, respectively, which is similar to the trends noted in the United States [3–10]. In the era of globalization and evidence-based medicine, cardiologists in Taiwan regularly read American journals and attend meetings in the United States; thus, the practice behaviors of these cardiologists would certainly have been influenced by the recommendations of crucial US trials and guidelines.

However, we could not rule out the possibility that the decline in the proportion of nonacute indication PCIs might be a result of physicians’ upcoding of the severity of diagnoses to justify the appropriateness of performing PCI. One US study has indicated that providers become increasingly aware of the clinical determinants of PCI appropriateness, and the pressure to reduce inappropriate use of PCI mounts may motivate physicians to initiate antianginal medications immediately before PCI, without an adequate interval to assess for response, or they may upcode patient-reported symptoms to influence apparent appropriateness [17]. Moreover, cardiac biomarkers, particularly cardiac troponin I, were used more frequently after 2003. The changing biomarker use is likely to have increased the detection rate of smaller infarcts and thus have predominantly influenced the rates of NSTEMI detection in Taiwan over time, suggesting that the expanded use of troponin testing may artificially alter the incidence rates of acute myocardial infarction and the coding of acute indications for PCIs [12].

Despite the overall decline in the proportion of PCIs for nonacute indications, regional and hospital variations still existed with the extent of decline in the percentage of nonacute indication for PCIs performed both in 2007 and 2012. Obviously, PCI practice patterns vary across regions (Fig. 2). The unique nonacute indication PCI practice profile of each region in Taiwan connotes the concept of a “surgical signature,” proposed by John Wennberg. This signature denotes a unique pattern for the utilization of common surgical procedures in different hospital referral regions [18]. In each regional signature, the rates of some procedures exceed the state (or national) average, whereas other procedures fall below the average. Contrasting signatures are noted in the Taipei and South regions (Fig. 2). In 2012, the percentage of nonacute indication PCIs was higher than 35% in 5 of 21 hospitals in the Taipei region and in none in the South region. In addition, 3 of 21 hospitals in the Taipei region showed a percentage change larger than 10%, but no hospitals in the South region showed a similar large increasing trend.

One possible explanation of the regional “PCI signature” is that local cardiovascular interventionists shared similar opinions on patient selection for PCIs. After the observation of a contrasting “surgical signature” between Boston and New Haven in the 1980s [19], Wennberg interviewed physicians in both regions and learned that “the low rate of carotid endarterectomy rates in New Haven compared with that in Boston could be attributed to a group of skeptical neurologists who simply did not believe in the procedure, preferring aspirin to surgery for any patient who came to them for advice. By contrast, the physicians in Boston had, on average, greater ‘faith’ in carotid artery surgery (although relative to many other parts of the country, the Boston rates were rather low). The conservative medical management of coronary artery disease and symptoms of menopause were more popular in Boston, whereas clinicians in New Haven more often preferred surgical management, which meant that more CABG and hysterectomies were performed. On the other hand, New Haven physicians were more enthusiastic about the conservative management of arthritis of the hip [18, pp. 58-59].”

From the point of view of professional organizations and insurance payers, the identification of hospitals that did not follow the general decreasing trend of hospitals with relatively high proportions of nonacute indication PCIs is important. In 2012, 5 hospitals showed percentage differences of larger than 10% and 6 showed nonacute indication PCI percentages higher than 35%; most of these were hospitals with high volumes in the Taipei region. One possible explanation was that many patients with difficult nonacute indications were referred to these high-volume hospitals from moderate- or low-volume hospitals. Another possible explanation was the high concentration of hospitals in the Taipei region, which led to sharing of the amount of acute indication cases, thereby resulting in a compensatory increase in the proportion of nonacute indication PCIs.

With regard to some hospitals showing trends that were contrary to general trends, Bradley et al. assessed the appropriateness of PCIs performed in Washington State and noted that hospitals in the tertile with the least improvement in the proportion of inappropriate PCIs had a temporal increase in the proportion of inappropriate PCIs from 12% in 2010 to 20% in 2013 [9]. The authors suggested that additional qualitative studies on hospitals with increasing proportions of inappropriate PCIs may serve to validate the patient selection processes.

The strengths of this study were the nationwide population-based design, which included all PCI cases in Taiwan and used strict inclusion criteria for acute indications to define nonacute indications. Nevertheless, our findings should be interpreted in light of the following limitations. First, information on the severity of symptoms, functional assessment, and results of stress testing were not available in the claims data; therefore, we could not accurately assess the indications and the appropriateness of performed PCIs.

Second, only 5 discharge diagnoses were available in the inpatient claims data released for research, and the data of some patients with acute indication ICD-9-CM codes might have been recorded in the sixth or later columns of the discharge diagnosis section. These cases might have been misclassified as those with nonacute indications. In addition, some patients with heart failure might be chronic instead of acute. However, the number of these misclassifications is likely small because most acute indication-related diagnoses would be placed in the first 5 discharge diagnosis columns.

Third, as previously discussed, we could not rule out the possibility that PCI operators might have upcoded the severity of the diagnosis to justify the appropriateness of performing PCIs. Fourth, on the contrary, according to our previous study [12], some coders might have undercoded unstable angina, resulting in the misclassification of an acute indication as a nonacute indication.

## Conclusions

This study indicates that approximately four-fifths of the studied hospitals in Taiwan showed a decline in the proportion of PCIs performed for nonacute indications; this trend might have been a result of the publication of the COURAGE study in 2007 and the AUC guideline in 2009. It is plausible that Taiwanese cardiologists are influenced by crucial trials and guidelines published in the United States.

However, a small number of hospitals still did not show decreasing trends after 2007 and showed a relatively high percentage of indication PCIs in 2012. Professional organizations and insurance payers should assessment of the appropriateness of PCIs performed in these hospitals. Furthermore, it is important to remind the physician to carefully assess the risk of patients with stable coronary disease before performing PCI. A systematic review suggests that ejection fraction, sex, diabetes mellitus, previous MI, and CRP independently predicted an increased risk of cardiovascular events and can help identify patients in need of further angiographic investigation [20].

## Abbreviations

AUC: Appropriate Use Criteria for Coronary Revascularization
CABG: coronary artery bypass grafting
COURAGE: Clinical Outcomes Utilizing Revascularization and Aggressive Drug Evaluation
ICD-9-CM: International Classification of Disease, Ninth Revision, Clinical Modification
NHI: National Health Insurance
NSTEMI: non-ST-segment elevation myocardial infarction
PCIs: percutaneous coronary interventions
SIHD: stable ischemic heart disease
STEMI: ST-segment elevation myocardial infarction
